# White-Light Emission of Dye-Doped Polymer Submicronic Fibers Produced by Electrospinning

**DOI:** 10.3390/polym10070737

**Published:** 2018-07-04

**Authors:** Monica Enculescu, Alexandru Evanghelidis, Ionut Enculescu

**Affiliations:** Group of Functional Nanostructures, National Institute of Materials Physics, Atomistilor 405A, P.O. Box MG-7, 077125 Magurele-Bucharest, Romania; alex.evanghelidis@infim.ro (A.E.); encu@infim.ro (I.E.)

**Keywords:** electrospinning, dye-doped polymer, submicronic fibers, white-light emission, tunable luminescence

## Abstract

Lighting and display technologies are evolving at tremendous rates nowadays; new device architectures based on new, microscopic building blocks are being developed. Besides high light-emission efficiencies, qualities including low cost, low environmental impact, flexibility, or lightweightness are sought for developing new types of devices. Electrospun polymer fibers represent an interesting type of such microscopic structures that can be employed in developing new functionalities. White-light-emitting fiber mats were prepared by the electrospinning of different dye-doped polymer solutions. Two approaches were used in order to obtain white-light emissions: the overlapping of single-dye-doped electrospun fiber mats, and the electrospinning of mixtures of different ratios of single-dye-doped polymer solutions. Scanning electron microscopy (SEM) was used to investigate the morphologies of the electrospun fibers with diameters ranging between 300 nm and 1 µm. Optical absorption and photoluminescence (PL) were evaluated for single-dye-doped submicronic fiber mats, for overlapping mats, and for fiber mats obtained from different compositions of mixtures. Depending on the ratios of the mixtures of different dyes, the luminance was balanced between blue and red emissions. Commission Internationale de L’Eclairage (CIE) measurements depict this fine-tuning of the colors’ intensities, and the right composition for white-light emission of the submicronic fiber mats was found.

## 1. Introduction

The electrospinning technique is a simple procedure to obtain polymeric and subsequently metallic, semiconducting, or ceramic fibers from solutions or melts [1,2,3,4]. The process was proven to be a low-cost, flexible, and scalable method to produce microfibers and nanofibers. Differently arranged (randomly or aligned) fibers with various morphologies can be obtained depending on the geometry of the electrospinning set-up [5,6,7,8]. A fine control of the electrospinning process parameters consequently results in a fine-tuning of the fibers’ properties [9,10], such as dimension, morphology (including nanostructuring), and composition (including doping with different compounds). 

Polymer microfibers and nanofibers produced by the electrospinning technique are in high demand because of their multiple applications, particularly in the biomedical field [11,12,13,14,15,16]. Besides medical applications, the polymer electrospun fibers are used also for filters [17,18], for cosmetics [18], in the clothing and textile industry [19,20], for electrical conductors [21], and for sensor devices [22]. For optical applications, such polymeric fibers are used for ultrafast photonics [23], for random lasers [24], for up-conversion [25], for white-light-emitting diodes [26], for dye-sensitized solar cells [27], for their nonlinear properties [28], and for optical fibers [29].

Functionalization of the polymer nanofibers may be done using nanoparticle addition or further chemical or electrochemical deposition of various compounds on the surface of the electrospun nanofibers [21,30,31]. Bundles of such nanofibers were proven to be able to mimic human muscle movements [32,33].

Interesting optical properties of the polymer fibers may be achieved by the electrospinning of dye-doped polymeric solutions [34,35]. Dyes are fluorescent chemical compounds that can re-emit light upon light excitation. Typically, they contain several combined aromatic groups or planar or cyclic molecules [36]. Dyes are commercially available and have been used in many applications, particularly in spectroscopy and medicine. Fluorescent dyes are mainly used in life sciences for non-destructive tracking or analysis of biological molecules [37,38], but they find a wide use in the industry for textile dyeing and as optical brighteners in laundry detergents [39,40], advanced cosmetic formulations [41], safety equipment and clothing, fine arts and design (posters and paintings) [42], synergists for insecticides, and experimental drugs [43]. Organic light-emitting diodes (OLEDs) and solar panels that collect more light (wavelengths) also have a more commercially available use nowadays. Dye-doped polymers have been studied in detail from the point of view of emission tuning with morphology and with composition [44,45,46]. 

By combining the wavelengths of dyes’ emissions that cover the entire visible spectral domain, white-light-emitting fibers can be produced. Up to 19% of worldwide electricity is consumed by lighting; therefore, increased efforts are being made to reduce the electricity dedicated to illumination, such as through the race to replace incandescent bulbs with less-energy-consuming LED lights [47,48,49]. Light adjusts people’s daily rhythms, and improving lighting can improve people’s well-being (at home) and productivity (in the workplace) [50]. In order to understand how to make this improvement, we have to understand how the human eye and light work. Because humans are trichromats (having in the middle part of the retina three types of cones—the color receptor cells: blue, green, and red), the perceived colors from the visible spectrum are made by mixing various percentages of blue, green, and red [51].

Sunlight has been researched since the beginning of optical science, and numerous efforts have been made for its standardization [52]. The complexity of daylight does not reside only in the mixture of the various colors, but also in the fact that there are changes in the intensities of different components depending on the season, month, place, and even time of day; that is, the morning hours’ light is an intense light suited for working, containing an enhanced blue-green component, while the light at dawn is a soothing light with an increased orange-red component. Thus, although commercial LEDs emit differently from sunlight, we perceive white light (of different types) when we use artificial lighting [53].

White-light-emitting low-dimensional structures have a wide range of applications, ranging from spectroscopic lab-on-a-chip sensing devices to IT and communications [54,55]. Numerous types of low-dimensional structures possessing white emission have been developed using different approaches and methods of preparation [56,57]. One can differentiate between these structures either by type of materials (ranging from polymers to oxides or composites) or by morphology and dimensions (ranging from zero-dimensional quantum dots to quasi one-dimensional fibers or complex-shaped structures) [58,59]. By tuning the composition and shape, one can easily define an application field in which these intrinsic properties can be exploited. 

The aim of this study is to present a simple and straightforward method to produce white-light-emitting structures. Thus, electrospinning was employed to fabricate submicrometric dye-doped polymer fiber mats. The photoemission of these submicronic fiber-based architectures could be tuned depending on the mode in which the doping was performed. As a consequence of the intensities’ values corresponding to each dyes’ emission maxima in the visible spectral domain, different types of white lighting could be obtained, including sun-like distributions. Two approaches were studied and are described in the paper, mats obtained by combining three single-dye-doped types of fibers and mats of fibers obtained by electrospinning mixtures of solutions doped with several dyes simultaneously. In both cases, high luminescence tunability was obtained, and white light was measured. The luminescent structures obtained in this way possess a specific design that can be employed in a wide range of applications in which one can exploit the fibrous structure of the building blocks, ranging from biological probes to high-surface-area/low-cost lighting devices. Being based on polymer fibers, the structures have the advantages of being lightweight and highly flexible, enabling their integration into different types of devices for which these characteristics are sought. The electrospinning technique allows such fibers to be fabricated simply and inexpensively and ensures they are readily scalable. Moreover, the structures are all organic, with low environmental impact.

## 2. Experimental Details

For obtaining the dye-doped polymer submicronic fibers, we used poly(methyl methacrylate) (PMMA) with a Mw of 300,000 and chemical formula of (C_5_O_2_H_8_)*n* at a concentration of 12.5 wt %. Dimethylformamide (DMF; anhydrous, 99.8%) was chosen as the solvent. The polymer solution was prepared at room temperature and stirred overnight. Blue-green- and orange-red-emitting dyes were used as dopants in order for the emitted lights’ wavelengths to cover a large area within the visible spectrum. Thus, the polymer solutions were doped with coumarin 7 (C7), coumarin 6 (C6), fluorescein (F), rhodamine 6G (Rh 6G), sulforhodamine B (SRh B), and sulforhodamine 101 (SRh 101). The chemical formula and optical properties of each dye used for doping are listed in Table 1. The dyes had 10^−3^ M concentrations in the polymer solutions. All chemical compounds were purchased from Sigma-Aldrich (St. Louis, MO, USA) and were used without any further purification.

During the fibers’ production through the electrospinning process, the fluid was pumped at a rate of 0.5 mL/h through a syringe with an automatic pump (New Era Pump System). The syringe needle was positively charged using a high-voltage supply (DC power supply: 30 kV Spellman SL300, New York, NY, USA). The resulting electric field caused solution jets to be pulled from the end of the droplet formed on the needle tip (the droplet formed a Taylor cone under the influence of the electric field) reaching, as fibers, the grounded metal collector consisting of a copper frame. 

**Figure 1:**
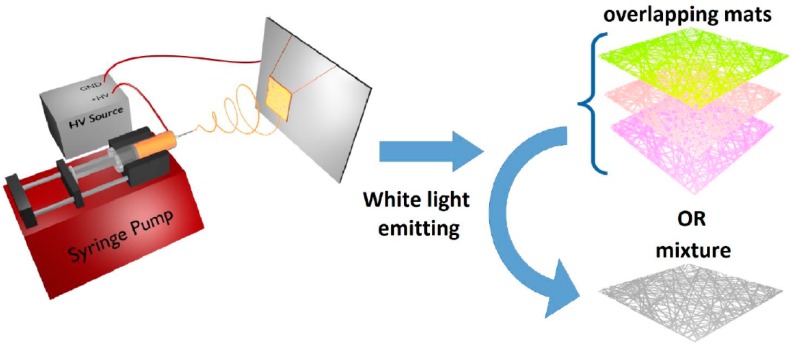
Schematic illustration of the experimental setup used for electrospinning of the dye-doped polymer solutions.

The electrodes were positioned at distances between 15 and 20 cm. The high voltage of 20 kV produced the electrically charged jet of the polymer solution, which dried and solidified into a polymer fiber along the trajectory towards the collector. Using high voltages resulted in the drawing of very fine fibers (submicrometric or even nanoscale) from a liquid (polymer solution or melt) with high aspect ratios and with the fiber diameter inversely proportional to the applied voltage. The set-up used to produce the dye-doped polymer fibers is illustrated in Figure 1.

Imaging of the solutions and fiber mats collected on copper frames was done at a 365 nm wavelength using a UVIlite lamp LF106L (Cambridge, UK) (6 W, filter 50 × 150 mm, 700 µW/cm^2^).

The characterization of the dye-doped polymer submicronic fibers from the point of view of structure and morphology was performed using scanning electron microscopy (SEM) (ZEISS EVO 50 XVP). For SEM imaging, small pieces of the mats were placed on sticky carbon discs. Because of the method used, the density of the fibers observed in the lower-magnification images differed from one sample to another. Prior to the imaging using the scanning electron microscope, the samples were sputtered with a thin layer of gold using an ANATECH Hummer VI DC sputtering system.

In order to evaluate the optical properties of the electrospun nanofibers, the absorption and transmission spectroscopy measurements were made using a UV-vis-NIR CARY 5000 spectrophotometer, and the photoluminescence (PL) was evaluated using a FLS920 EDINBURGH INSTRUMENTS spectrofluorimeter (equipped with a 450 W Xe lamp and double monochromators in both excitation and emission). The chromaticity measurements were made using a Minolta CS-2000 spectroradiometer (Konica Minolta, Tokyo, Japan).

## 3. Results and Discussion

### 3.1. Single-Dye-Doped Polymer Fibers

**Figure 2:**
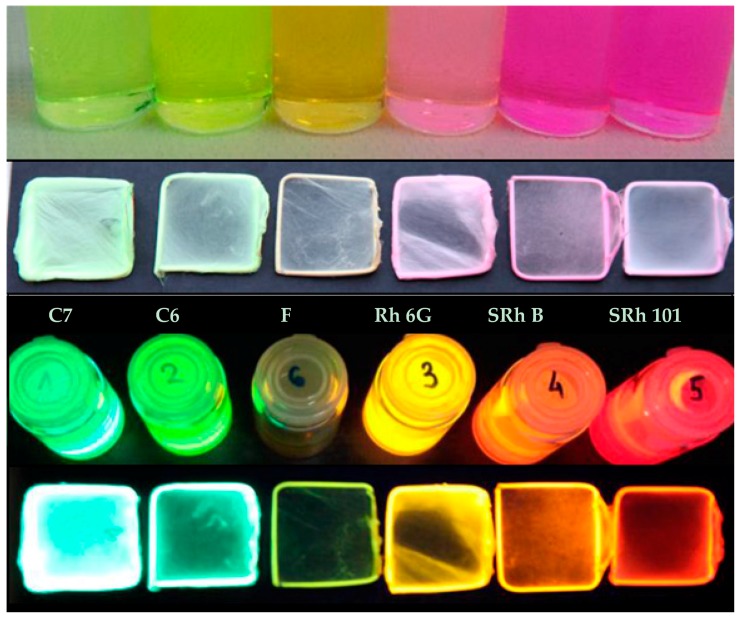
Images of the polymer solutions and the corresponding electrospun submicronic fiber mats collected on copper frames in visible light (**upper**) and UV light (**lower**).

Submicronic fiber mats were produced using copper frames as collectors during the electrospinning process. Images of the single-dye-doped polymer solutions used for the electrospinning method and their corresponding self-supporting electrospun fiber mats under either visible or UV light are presented in Figure 2.

There are many parameters that have to be controlled during the electrospinning process. One has to control the ambient parameters (temperature, humidity, air currents, etc.), the solution parameters such as the polymer concentration (MW and solvent), and also the set-up parameters (the collector geometry that results in different fiber alignments, the distance between electrodes, the applied voltage, the flow rate, and the needle diameter). By varying these parameters, the tailoring of the fibers’ morphological properties (diameter, texture, and pattern) becomes possible.

The PMMA matrix was chosen as the carrier polymer because the hydrophobic properties of PMMA would ensure the fibers’ integrity from both morphological and optical points of view. Besides its favorable optical properties, our experiments have shown that PMMA also offers the mechanical strength necessary for the collection of suspended fibers with submicronic diameters and lengths on the centimeter scale (sample size was 3 cm × 3 cm).

**Figure 3:**
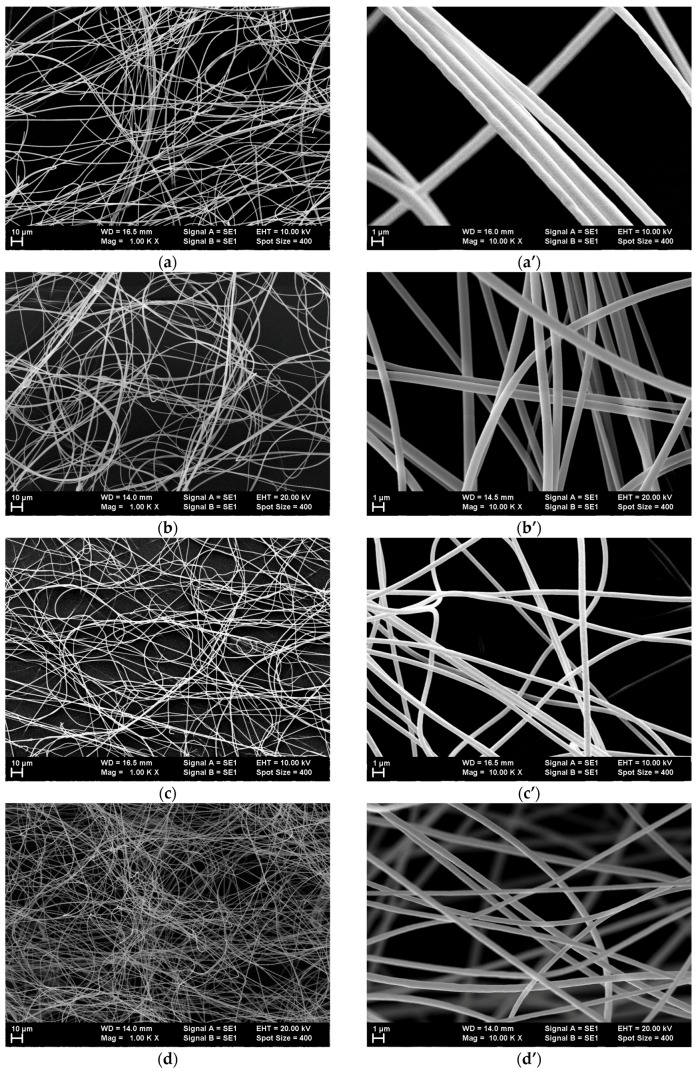

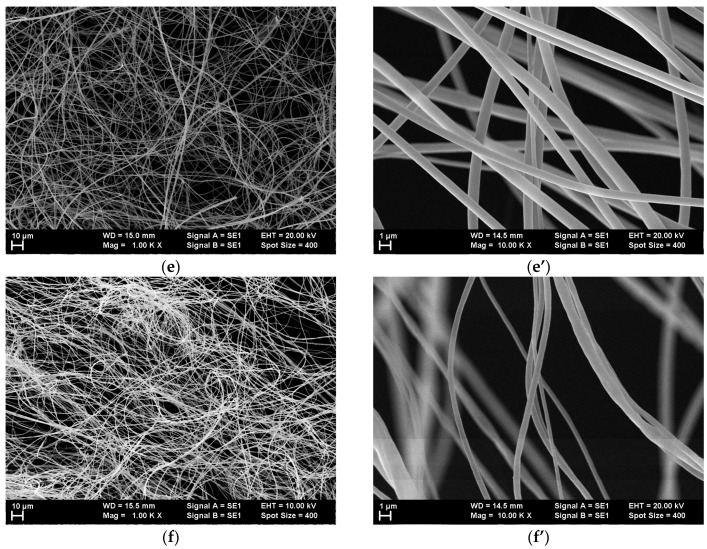
Scanning electron microscopy (SEM) images at two different magnifications (1000× and 10,000×) of the electrospun polymer fibers doped with (**a**,**a’**) coumarin 7, (**b**,**b’**) coumarin 6, (**c**,**c’**) fluorescein, (**d**,**d’**) rhodamine 6G, (**e**,**e’**) sulforhodamine B, and (**f**,**f’**) sulforhodamine 101.

Lower- and higher-magnification SEM images of the single-dye-doped polymer fibers produced by electrospinning are presented in Figure 3 for all the dyes that were used. The lower-magnification SEM images obtained at 1000× illustrate the random placement of the nanofibers and the uniformity of the diameters’ distributions, which are difficult to observe in the higher-magnification SEM images.

The fibers’ diameters ranged between 300 nm and 1 µm.

The thicknesses of the electrospun mats are very important parameters, particularly for the intensities of absorption and luminescence. However, it is difficult to precisely measure such thicknesses because of the fact that the electrospun mats are fluffy (aired), containing lots of empty space, and because their thicknesses cannot be assimilated with the thicknesses of a certain number of overlapped fibers.

Because of the way an electrospun fiber mat is formed in the optically transparent regime (i.e., at low fiber densities), it is difficult to define a meaningful geometrical thickness, as up to 90–95% of the mat can be empty space. This is further complicated by the flexibility of the fibers, which makes the mats compressible in an uncertain way. 

However, given the optical nature of the application at hand, a relevant characteristic is the optical transmission of the fiber mats in the visible range. This is directly linked to the true geometrical thickness but is far easier to measure. As it depends on the fiber density, this “thickness” can be controlled by varying the collection time. All the samples were produced using the same collection time, which means that they should have had approximately the same transmission values and fiber densities. 

In order to demonstrate the similarity of the samples regarding the measured thicknesses, we evaluated the transmission spectra in the interval 600–800 nm in which the transmittance could be compared, because in this wavelength interval, there were no absorption bands of the dyes. 

Information regarding the optical transmission values of the samples are presented in the Supplementary Materials, Figure S1.

**Figure 4:**
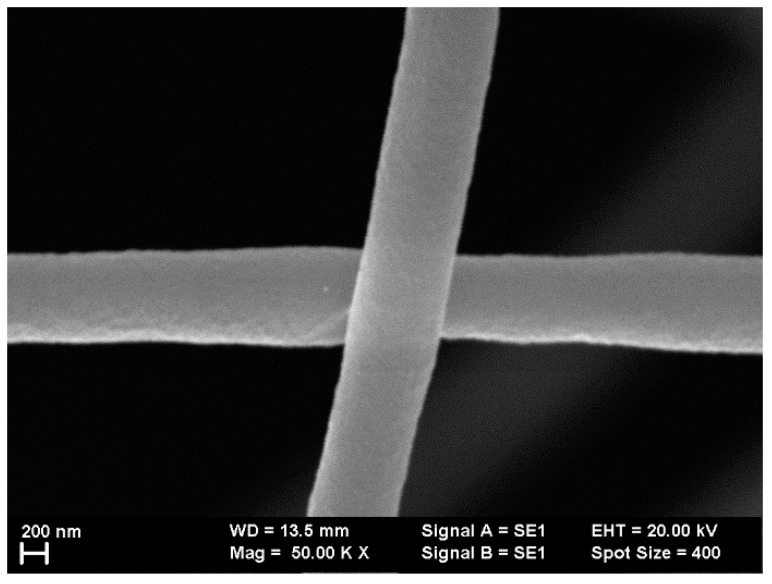
Scanning electron microscopy (SEM) image presenting the surface uniformity for rhodamine 6G (Rh 6G)-doped polymer fibers.

Previous studies have shown that the presence of different dyes in polymer solutions leads to changes in the morphologies of the electrospun nanofibers as a result of the different sizes of dye molecules [45]. In order to avoid fabricating fibers with different morphologies, the concentration of the polymer in the solution was increased compared to prior investigations [44,45]. Thus, for a 12.5 wt % concentration of PMMA in DMF, uniform submicronic fibers were obtained by electrospinning for all single-dye-doped polymer solutions. It could be observed that all the fibers had smooth surfaces (e.g., surfaces of Rh 6G-doped fibers are presented in Figure 4). Rh 6G was chosen as an example because its molecule has an average size when compared with the other dyes used in this study.

**Figure 5:**
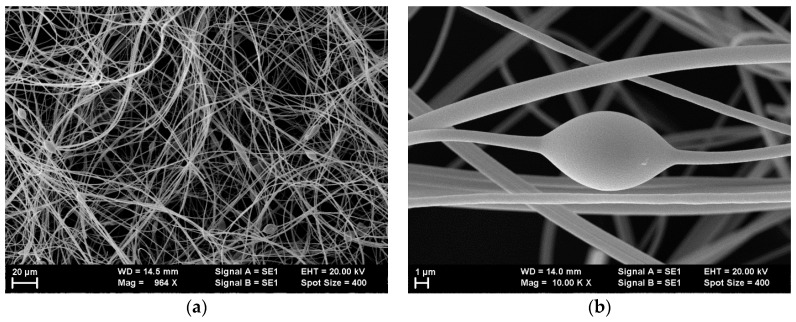
Beads-on-string morphology observed for coumarin 6 (C6) sample: 1000× (**a**), and 10,000× (**b**).

However, for the C6-doped polymer fibers, we observed the rare occurrence of a beads-on-string morphology (Figure 5), likely formed at the beginning of the electrospinning process during the period when a stabilization of the process takes place. The appearance of such a specific polymer fiber morphology for the electrospinning of the C6-dye-doped solution is consistent with the previous studies [45].

The appearance of beads on fibers is strongly related to the viscosity and surface tension (among other parameters) [60,61]. In the C6-doped PMMA fibers, the appearance of beads was likely related to the intrinsic properties of C6, leading to the highest probability for the occurrence of such morphology. It is well known that C6 fluorescence in solvents depends critically on concentration and on the pattern of substitution for the amine group [62]. Relatively large shifts in both absorption and fluorescence were reported not only in solutions but also in polymers [63]. C6 is known for its high degree of sensitivity to its local environment, which may result in alterations to the morphological and optical host’s properties, including viscosity [64,65]. Thus, the presence of C6 may have affected the viscosity and the surface tension of the polymer solution more so than for the other dyes, leading to the rare appearance of beads during the electrospinning process.

**Figure 6:**
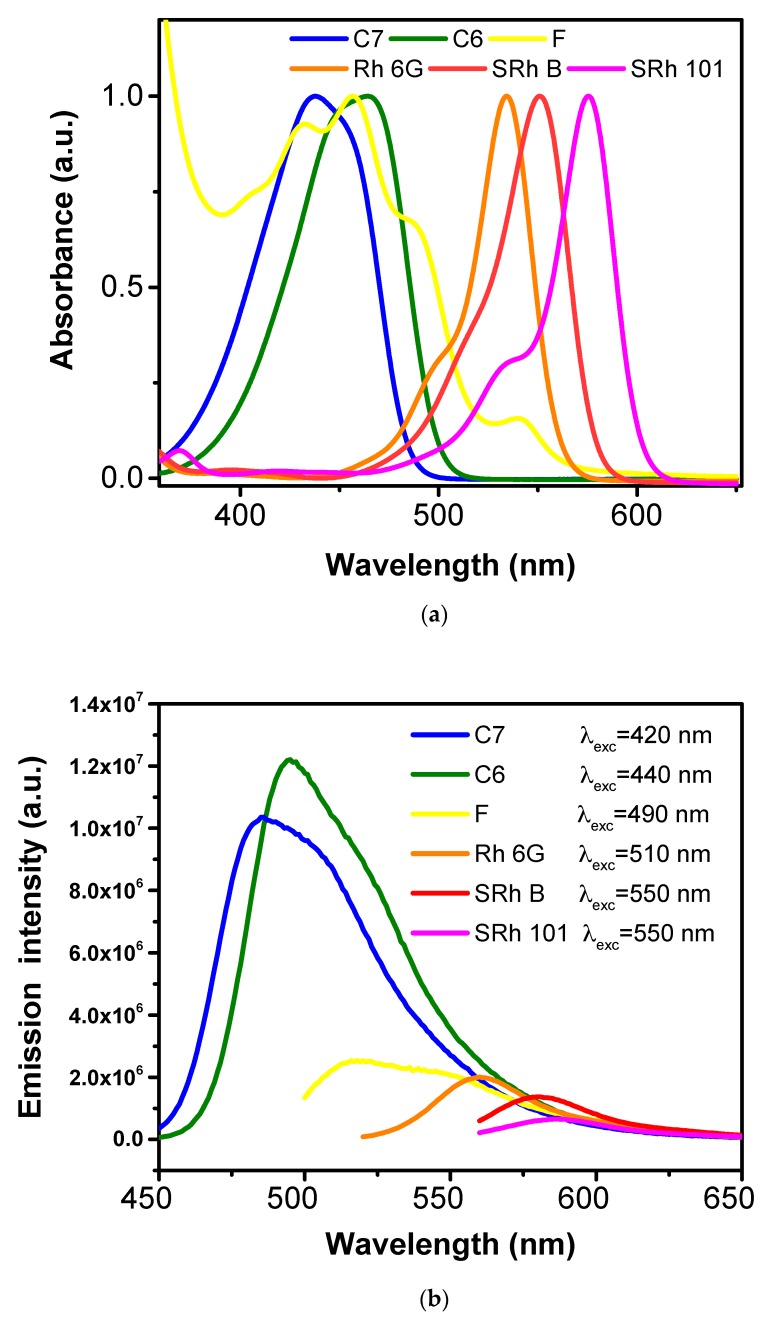
Absorption (**a**), and photoemission (**b**) spectra of single-dye-doped polymer fibers.

The optical absorbance and PL spectra of the single-dye-doped fiber mats were measured and plotted, and the results are presented in Figure 6a,b. 

As expected, the optical absorptions of the single-dye-doped fiber mats covered the spectral domain 400–600 nm with increasing intensities from the blue region towards the red region, while the emissions covered the spectral domain 450–650 nm with higher intensities in the blue-green region and significantly lower intensities of the emission in the orange-red region of the visible spectra. The decreasing of the PL intensities was in total agreement with studies from the literature related to the efficiencies of the dyes when dissolved in different solvents [66,67,68]. There is a strong relation between PL and the molecular structure of dyes, indicating different efficiencies. This further leads to different PL intensities, although the dyes had the same concentration (10^−3^ M) in the solution of PMMA (12.5 wt % in DMF). 

Depending on the conditions and solvent, fluorescein may dimerize. A dimer–monomer equilibrium appeared, thus leading to the appearance of different peaks in the absorption spectra [69]. Moreover, different forms (cationic, neutral, anionic, and dianionic) of the fluorescein’s molecule may be found in a dye-doped solution [70]. Therefore, we may observe more peaks in both absorption and emission spectra of a F-doped polymer fiber mat. Absorption peaks appeared also at wavelengths of less than 350 nm, in the UV region of the spectrum.

The 420 nm excitation with C7-doped fibers revealed the overlapping of two peaks appearing at 485 and 505 nm, whereas for C6-doped fibers, two peaks at 495 and 515 nm appeared under 440 nm excitation. The appearance of the shoulder in PL may have been due to the protonated form of the dye, which was previously reported for coumarins [66]. The 490 nm excitation with F-doped fibers revealed a broad PL spectrum formed by an overlapping of peaks between 515 and 575 nm. The Rh 6G-doped fibers exhibited a peak at 560 nm under 510 nm excitation, while under 550 nm excitation, the SRh B-doped fibers presented a PL peak at 580 nm and the SRh 101-doped fibers presented an emission peak at 590 nm.

**Figure 7:**
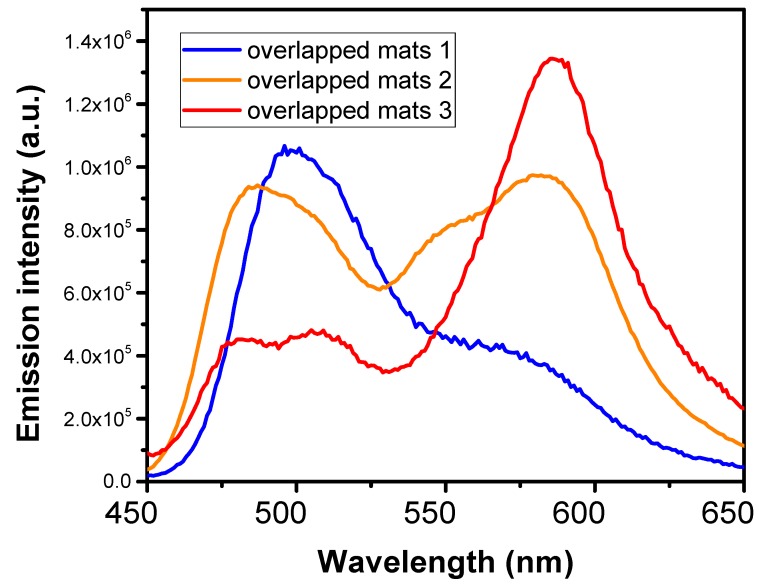
Photoluminescence (PL) spectra of overlapping mats of coumarin 6, rhodamine 6G, and sulforhodamine 101 doped fibers with different thicknesses.

Next, the emissive properties of the overlapping single-dye-doped fibers’ layers were evaluated. By varying the thickness of the layers, we were able to tune the emission spectra of the overlapped fiber mats and thus able to enhance the blue and red contributions to the spectra or to obtain a balanced combination of the peaks. The downside of such an approach is that there are a limited number of layers that can be used and that too many layers overlap, further resulting in the obstruction of light and the screening of the first layers’ emissions. PL excited at 420 nm for different overlapping combinations of three single-dye-doped fiber mats, C6, Rh 6G, and SRh 101, are presented in Figure 7. 

The volumes of the dye-doped solutions electrospun for the different overlapping fiber mats were varied, leading to a variation in the thicknesses of the layers, until a balanced PL spectrum was achieved. Thus, the balance in sample 2 was achieved by overlapping C6, Rh 6G, and SRh 101 mats in a 1/5/10 ratio, while sample 1 was formed by overlapping C6, Rh 6G, and SRh 101 submicronic fibers in a 10/5/5 ratio and sample 3 was formed by overlapping C6, Rh 6G, and SRh 101 submicronic fibers in an equal 10/10/10 ratio.

### 3.2. Dye-Doped Polymer Fibers Obtained from Mixed Solutions

Copying the complexity of white light requires a more versatile approach; therefore, we used mixed single-dye-doped polymer solutions in different percentages in order to be able to tune the emissions of the electrospun fiber mats.

Table 2 presents the whole range of mixed solutions used for testing the emissive properties of the polymer fiber mats. Nine mixtures were prepared using single-dye-doped polymer solutions. Mixtures a, b, and c were prepared without the F solution, and a pronounced gap appeared in the PL spectra in the range 520–540 nm (observed in Figure 7 for overlapping mats). Therefore, although having a complicated overlapping of peaks in the PL spectrum due to the presence of various aggregates, as previously explained, fluorescein was found to be a good solution for leveling the spectrum in the yellow region. Thus, six additional mixtures containing six different dye-doped polymer solutions in different ratios (including fluorescein) were prepared. The spectra of mixtures d, e, and f were only slightly different from the spectrum of mixture 3. Therefore, only three mixtures were chosen to be further analyzed by optical and morphological measurements, namely, mixtures 1, 2, and 3, which illustrate the possibility of balancing the intensities between red and blue emissions.

**Figure 8:**
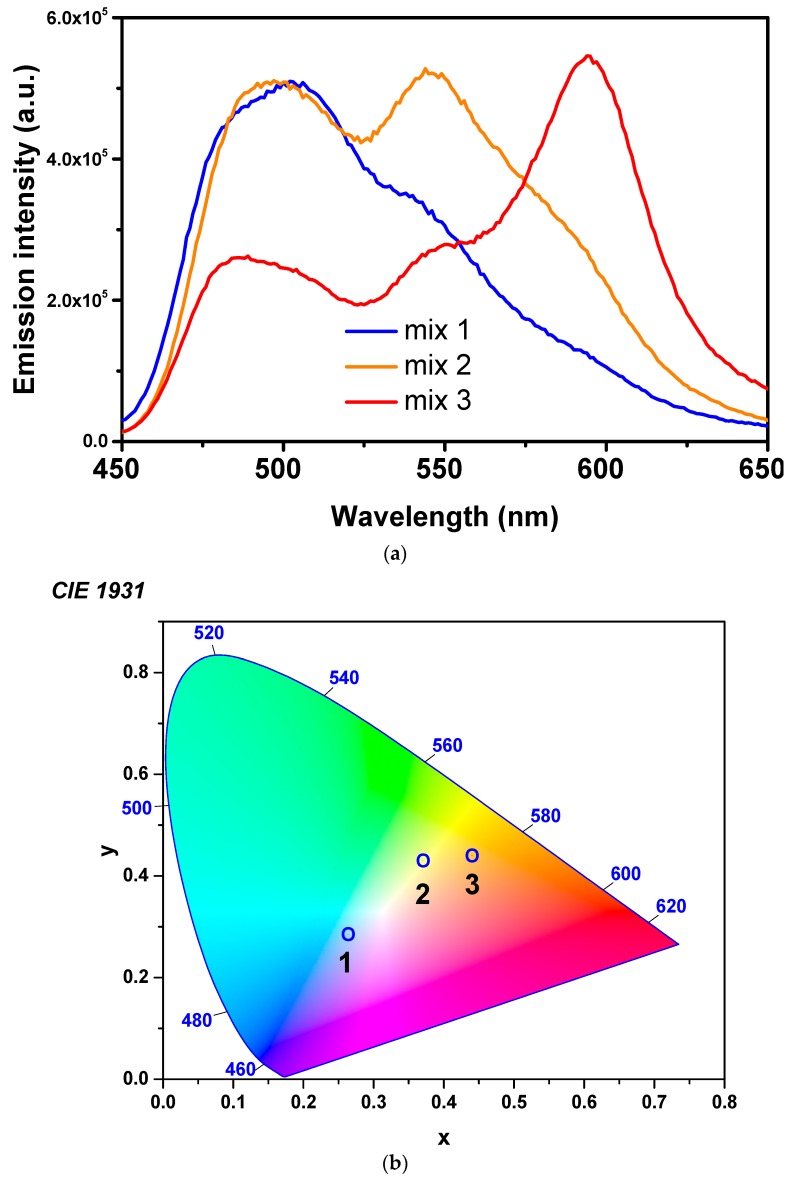
(**a**) Photoluminescence (PL) spectra of submicronic fibers produced by electrospinning from different mixtures of dye-doped polymer solutions, and (**b**) their corresponding coordinates on Commission Internationale de L’Eclairage (CIE) 1931 color space chromaticity diagram.

PL spectra of the fiber mats produced by the electrospinning of different combinations of single-dye-doped polymer solutions (mixtures 1, 2, and 3) are presented in Figure 8a. The spectra illustrate the possibility to balance the luminescence between blue and red emissions, depending on the ratios of the different dye-doped solutions. Commission Internationale de L’Eclairage (CIE) coordinates of the fibers obtained by electrospinning of different mixtures are presented in Figure 8b. The CIE measurements depicted the fine-tuning of the intensities of colors emitted by the fiber mats composing the white light. Thus, the emissions of the submicronic fibers obtained from the C7/C6/F/Rh6G/SRhB/SRh101 mixtures had the CIE coordinates of (0.27, 0.29) for the composition 10/20/20/20/10/20 (mixture 3), (0.38, 0.44) for the composition 15/30/30/10/7.5/7.5 (mixture 2), and (0.44, 0.44) for the composition 12.5/25/40/7.5/7.5/7.5 (mixture 1).

**Figure 9:**
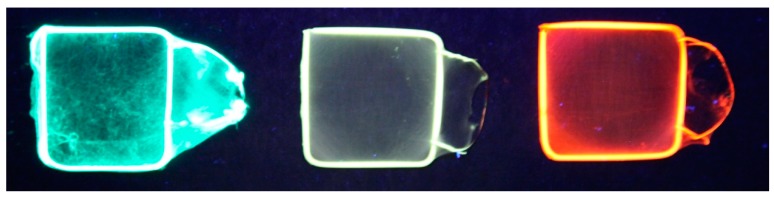
Blue-green (coumarin 7 (C7))-, white-light (mixture 2)-, and orange-red (sulforhodamine 101 (SRh 101))-emitting flexible and thin fiber mats under UV light excitation (365 nm).

The excitation wavelength for all the fibers obtained by the electrospinning of mixed solutions was 420 nm, corresponding to the excitation wavelength of C7, the dye with the smallest values for both the excitation and the emission wavelengths. The electrospinning of the mixture formed by C7/C6/F/Rh6G/SRhB/SRh101 in a ratio of 15/30/30/10/7.5/7.5 (mixture 2; presented in Figure 8a) resulted in a submicronic fiber mat that exhibited white-light emission. The UV image of the white-light-emitting fiber mat (mixture 2) compared with the green-emitting and red-emitting fiber mats is presented in Figure 9.

It could be observed both for the overlapping fiber mats and for the fiber mats obtained by electrospinning mixtures of single-dye-doped polymer solutions that for equal amounts of different dyes, the red contribution to the spectra had a higher intensity. The increased intensity of the red contribution appeared as a result of the fact that the red component was excited by both the wavelength of excitation and by the light emitted by the green-yellow components of the mixtures.

**Figure 10:**
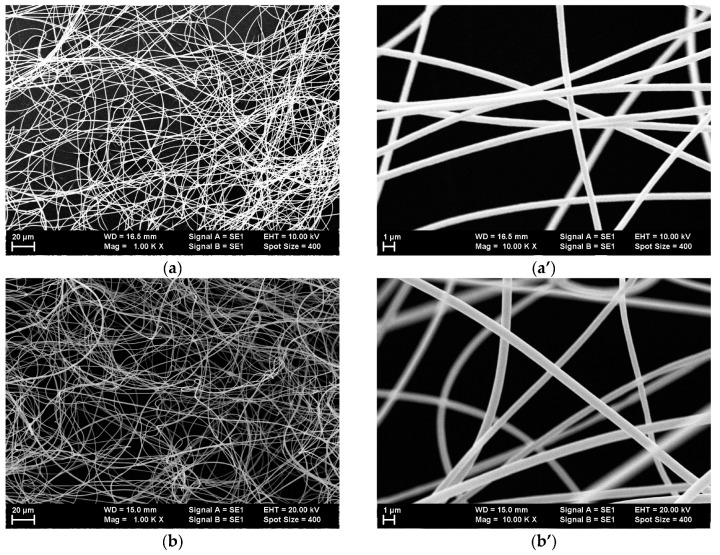

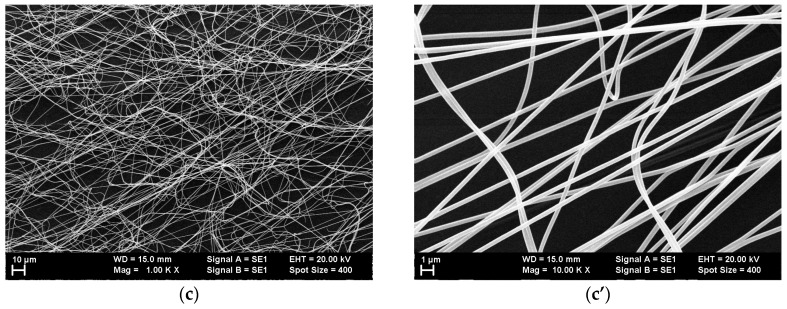
Scanning electron microscopy (SEM) images of submicronic fibers obtained by electrospinning of mixtures of single-dye-doped polymer fibers: (**a**,**a’**) mix 1; (**b**,**b’**) mix 2; (**c**,**c’**) mix 3.

Morphology studies have showed that electrospinning mixtures of dye-doped polymer solutions in different combinations results also in submicronic fibers with smooth surfaces, uniform dimensional distribution, and diameters comparable to those of single-dye-doped electrospun polymer fibers. The diameters of the fibers obtained by electrospinning mixtures of solutions also ranged between 300 nm and 1 µm. The SEM images of the submicronic fibers are presented in Figure 10.

The dye-doped polymer fibers produced by electrospinning presented similar optical properties over a long period of time. The PL of the electrospun fibers was very stable. The fibers showed identical PL signals after storage under normal atmospheric conditions. Surface enrichment of the dye is commonly observed in dye-doped polymeric systems [71]. The fibers may have presented a possible segregation of the dyes on their surfaces during their formation, but this was difficult to be confirmed experimentally because of the fact that the solidification of fibers takes place over a very short period of time.

## 4. Conclusions

We present a simple method to fabricate flexible, white-light-emitting nanofibers using the electrospinning methods of dye-doped polymers. Different mixtures of single-dye-doped polymer solutions were used in order to tune the emissions of electrospun nanofibers. The present study shows that electrospun nanofibers obtained from mixtures of single-dye-doped solutions present white-light emissions and may have potential applications as new light sources or sensory materials for smart textiles. White-light-emitting electrospun submicronic fibers were successfully prepared through the electrospinning of mixtures of single-dye-doped PMMA solutions in DMF using a single capillary spinneret. The emission intensities of colors composing the white light changed as the composition of the mixtures changed, increasing either the blue-green component or the orange-red component of PL. The white-light-emitting submicronic fibers obtained from the C7/C6/F/Rh6G/SRhB/SRh101 mixture with composition 15/30/30/10/7.5/7.5 had CIE coordinates of (0.38, 0.44). Our results show that white-light-emitting fibers can be produced through the use of multicomponent single-dye-doped PMMA mixtures.

## Figures and Tables

**Table 1:** Chemical formulas, molecular structures, and optical properties of the dyes used for doping the polymer solutions.

Dye	Chemical Formula	Molecular Structure	Absorption Wavelengths	Emission Wavelengths
Coumarin 7	C_20_H_19_N_3_O_2_	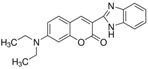	437 and 455 nm	485 and 505 nm
Coumarin 6	C_20_H_18_N_2_O_2_S	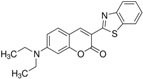	448 and 465 nm	495 and 515 nm
Fluorescein	C_20_H_12_O_5_	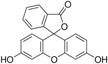	430, 457, and 487 nm	515, 550, and 575 nm
Rhodamine 6G	C_28_H_31_N_2_O_3_Cl	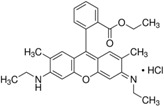	534 nm	560 nm
Sulforhodamine B	C_27_H_29_N_2_NaO_7_S_2_	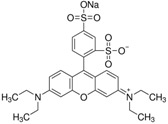	550 nm	580 nm
Sulforhodamine 101	C_31_H_29_ClN_2_O_6_S_2_	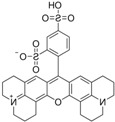	575 nm	590 nm

**Table 2:** Composition of dye-doped polymer solution mixtures.

Solution	Percentage of Single-Dye Solution					
	C7	C6	FL	Rh 6G	SRh B	SRh 101
Mix a	20	20	-	20	20	20
Mix b	16.66	33.33	-	33.33	8.33	8.33
Mix c	12.5	25	-	25	12.5	25
Mix 3	10	20	20	20	10	20
Mix d	12.5	22.5	22.5	22.5	10	10
Mix e	17	22.5	22.5	22.5	7.5	7.5
Mix f	5	25	25	25	5	5
Mix 2	15	30	30	10	7.5	7.5
Mix 1	12.5	25	40	7.5	7.5	7.5

## Supplementary Materials

The following are available online at http://www.mdpi.com/2073-4360/10/7/737/s1, Figure S1: Transmission spectra for the single-dye-doped polymer fiber mats produced by electrospinning.
