# Supplementary material for: White-Light Emission of Dye-Doped Polymer Submicronic Fibers Produced by Electrospinning
Source: Polymers (Basel). 2018 Jul 4;10(7):737. doi: 10.3390/polym10070737 (PMC6404093; doi:10.3390/polym10070737)
Supplement: Supplementary file 1 [file polymers-10-00737-s001.pdf]

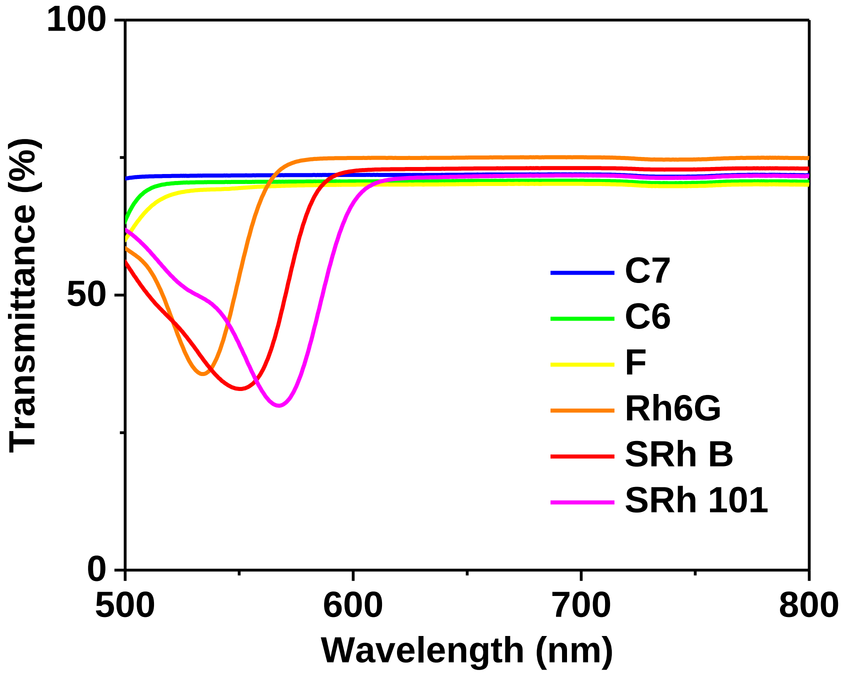

Figure S1. Transmission spectra for the single-dye doped polymer fiber mats produced by electrospinning.
